# Synaptic Characteristic of Hafnia-Based Ferroelectric Tunnel Junction Device for Neuromorphic Computing Application

**DOI:** 10.3390/nano13010114

**Published:** 2022-12-26

**Authors:** Wonwoo Kho, Gyuil Park, Jisoo Kim, Hyunjoo Hwang, Jisu Byun, Yoomi Kang, Minjeong Kang, Seung-Eon Ahn

**Affiliations:** 1 Department of IT Semiconductor Convergence Eng, Tech University of Korea, Siheung 05073, Republic of Korea; 2 Department of Nano & Semiconductor Eng, Tech University of Korea, Siheung 05073, Republic of Korea

**Keywords:** FTJ, synaptic devices, SNN, STDP, neuromorphic computing

## Abstract

Owing to the 4th Industrial Revolution, the amount of unstructured data, such as voice and video data, is rapidly increasing. Brain-inspired neuromorphic computing is a new computing method that can efficiently and parallelly process rapidly increasing data. Among artificial neural networks that mimic the structure of the brain, the spiking neural network (SNN) is a network that imitates the information-processing method of biological neural networks. Recently, memristors have attracted attention as synaptic devices for neuromorphic computing systems. Among them, the ferroelectric doped-HfO_2_-based ferroelectric tunnel junction (FTJ) is considered as a strong candidate for synaptic devices due to its advantages, such as complementary metal–oxide–semiconductor device/process compatibility, a simple two-terminal structure, and low power consumption. However, research on the spiking operations of FTJ devices for SNN applications is lacking. In this study, the implementation of long-term depression and potentiation as the spike timing-dependent plasticity (STDP) rule in the FTJ device was successful. Based on the measured data, a CrossSim simulator was used to simulate the classification of handwriting images. With a high accuracy of 95.79% for the Mixed National Institute of Standards and Technology (MNIST) dataset, the simulation results demonstrate that our device is capable of differentiating between handwritten images. This suggests that our FTJ device can be used as a synaptic device for implementing an SNN.

## 1. Introduction

Currently, the world is undergoing an exponential increase in annual data generation. This has been facilitated by technological developments, such as those in big data, artificial intelligence (AI), and autonomous vehicles. Specifically, unstructured data, such as voice and image data, are rapidly increasing [1]. The conventional von Neumann computing architecture is a serial processing method, in which the logic and memory are separated. Accordingly, a bottleneck phenomenon occurs, owing to the difference in the data processing speeds between the CPU and memory. In addition, there is a clear limit to the scalability of large-scale network applications, owing to the excessive power consumption when fetching in/out data for multiply and accumulate operations. Neuromorphic computing, which mimics the human brain, has been proposed as a solution to these issues [2]. The human brain consists of approximately 10^11^ neurons and 10^15^ synapses for performing high-density parallel processing [3] at a low power of 10 W [4]. The synaptic plasticity between neurons is used to characterize the strength of the synaptic connections. Adjusting the synaptic plasticity alters the synaptic weight and enables coordinated control of memory, computation, reasoning, and learning [5,6]. To implement an AI system, it is necessary to build a system with a structure similar to that of the brain [7]. As a result, there is growing interest in neural networks and their learning rules, as these can imitate how the human brain’s many neurons are connected by countless connections. Spike timing-dependent plasticity (STDP) is a learning rule for determining how the weight of a synapse changes in the neural network, that is, whether the synaptic plasticity dominates. It was initially proposed to conceptually imitate biological synaptic plasticity [8]. Since then, interest in spike-based neural networks has grown, as experimental evidence of STDP as an effective plasticity mechanism has emerged [9]. Accordingly, development of an artificial synaptic device that can apply STDP learning has also emerged as a significant challenge.

Although neuromorphic semiconductor integration is limited by physical and technical factors, the amount of data needed for AI learning continues to rise sharply each year [10]. Additionally, because the brain has more synapses than neurons, efforts to reduce the sizes of these synapses are unquestionably important, as they are in all artificial neural networks. As a possible solution, B. Widrow and M. Hoff created the memristor in 1960 as a reasonably straightforward transistor-like device for mimicking a synapse. However, the three-terminal device was not very effective [11]. In 2007, Snider proposed using a memristor comprising a two-terminal, nanoscale, reactive-switching device to mimic a synapse [12]. Generally, memristor-based neuromorphic technology is specifically suggested by numerous research groups to emulate biological synapses, as it can express values between 0 and 1 in analog form. Various memristive devices are being researched for use as synaptic devices, including ferroelectric-based memory, phase-change memory, magnetoresistive random-access memory, and resistive switching memory. Among them, there is much interest in ferroelectric tunnel junctions (FTJs) based on ferroelectric doped-HfO_2_. First, the FTJ offers low power consumption, owing to its tunneling mechanism. It also has excellent integration and resembles a biological neuron-synapse-neuron structure, owing to its straightforward two-terminal structure. These reasons make it useful for its application in three-dimensional, high-density, cross-point arrays. It also offers several advantages for an artificial synapse: non-volatility, analog switching, energy efficiency, scalability, linearity, symmetric synaptic weight updates, large dynamic range, fast operating speed, and small cycle-to-cycle variations [13,14,15]. Accordingly, it is considered as a strong candidate for a potential next-generation synaptic device for fulfilling a variety of requirements for synapse devices, such as the high-density formation of neuron–synapse network structures, complementary metal–oxide–semiconductor (CMOS) device/process compatibility, small device dimensions (<10 nm), and the implementation of many states with one pulse [16]. Therefore, we analyzed the synaptic properties of the HZO FTJ device learned using the SNN-based STDP as a synaptic device for neuromorphic computing applications. To implement the synaptic characteristics, atomic force microscopy (AFM, Park System) in a piezo-response force microscopy (PFM) mode was used to demonstrate the ferroelectricity, and a conductive AFM (C-AFM) mode and semiconductor parameter analyzer (Keithley 4200 with 4225-PMU module) both supported the FTJ mechanisms. We also altered the spike conditions (which significantly impact the STDP results), and improved the synaptic characteristics in accordance with the spike timing. A symmetric-non-linearity model was used to investigate the synaptic properties concerning symmetry and linearity. Based on the measured data, a CrossSim simulation was conducted using a small-image (8 × 8 pixels) dataset from the University of California at Irvine (UCI) and a large-image (28 × 28 pixels) dataset from the Mixed National Institute of Standards and Technology (MNIST). Ultimately, we determined that the HZO FTJ-based memristor is suitable as a synaptic device for an efficient SNN from the high accuracy of pattern recognition.

## 2. Materials and Methods

**Figure 1:**
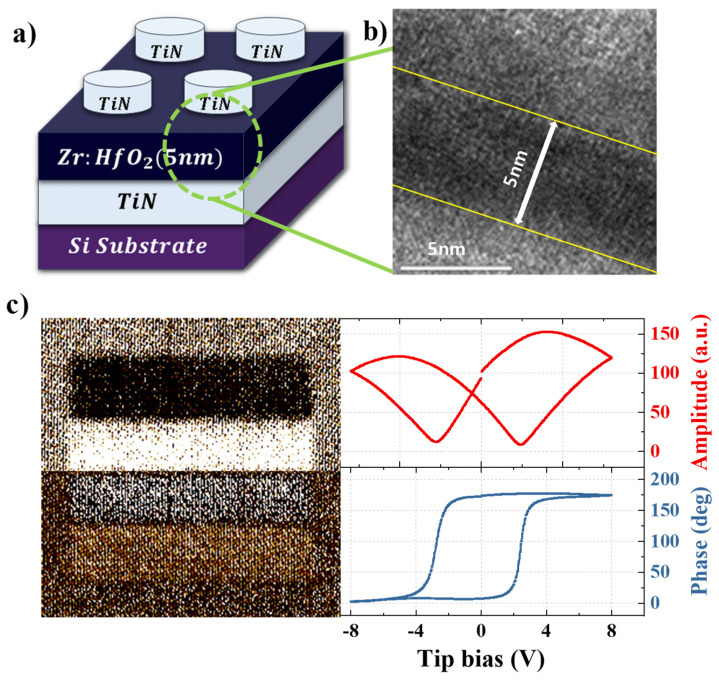
Schematic representation and polarization characteristic of HZO FTJ devices. (**a**) Structure of the TiN/HZO/TiN (metal-ferroelectric-metal (MFM)) FTJ device. (**b**) Cross-sectional transmission electron microscope (TEM) image of FTJ device. (**c**) Ferroelectric properties: PFM measurements of the amplitude (left, upper panel) and phase (left, lower panel) of the HZO/TiN structure after domain patterning with opposite polarities. Local ferroelectric properties: PFM amplitude (right, upper panel) and phase (right, lower panel) hysteresis loops of the HZO/TiN structure.

The HZO ferroelectric thin film was deposited using plasma-enhanced atomic layer deposition (iOV DX2 PEALD, iSAC Research, Republic of Korea) to create an FTJ device used to implement the artificial synaptic device. The ferroelectric layer was deposited on a TiN/SiO_2_/Si substrate using RF power at 180 °C. Tetrakis (ethylmethylamido)-hafnium (IV) and tetrakis (ethylmethylamido)-zirconium (IV) were used as precursors, and O_2_ was used as the oxidant. The HfO_2_ and ZrO_2_ were alternately deposited to prepare a 5 nm-thick HZO film at a ratio of Zr:Hf = 1:1. The top TiN electrode was deposited on the ferroelectric layer by radio frequency magnetron sputtering in an Ar and N_2_ atmosphere with a circular-patterned hard mask (r = 100 µm). Finally, by performing rapid thermal annealing at 600 °C for 60 s in a N_2_ environment, a HZO FTJ device with a metal-ferroelectric-metal (MFM) structure was created, as illustrated in Figure 1a. Figure 1b indicates a transmission electron microscope image of a cross-section of the stacked MFM structure. The ferroelectric layer with a thickness of 5 nm can be visually confirmed.

Measurement techniques and measuring equipment are as follows. Ferroelectric properties of the HZO ferroelectric thin films were performed using AFM (XE7, Park Systems, Republic of Korea) with lock-in amplifier (SR830 DSP, Stanford Research Systems, CA, USA). In current mapping for memory characteristics verification, an Ultra-Low Current Amplifier (ULCA, Park Systems) was used to amplify low-level currents. Images scanned by AFM were analyzed using image analysis software (XEI, Park Systems). All other electrical measurements were performed using a parameter analyzer (4200A-SCS, Keithley, USA) with a 4225-PMU. The low-level current was measured using a preamplifier connected to the SMU. All measurements were performed at room temperature.

## 3. Results and Discussions

The ferroelectricity was demonstrated based on AFM measurements. The experiment was conducted in the PFM mode in electrostatic force microscopy, which uses an electrostatic force to measure electrical properties. In the PFM mode, the conductive cantilever acts as the top electrode while scanning in contact with the sample (measured in the HZO/TiN structure). The local piezoelectrical strain is obtained as a response amplitude from an AC signal applied to the probe scanning the sample surface. It relates to the polarization magnitude and is represented as a piezoelectrical coefficient (d_zz_) [17]. Two effects need to be proven to demonstrate ferroelectricity: the presence of switchable polarization domains, and hysterical switching between opposite polarization states by an electric field. First, the region was partitioned and electrically polled to demonstrate the presence of a switchable polarization domain. By applying bias to the tip, it was possible to compare the before and after images of the polling as follows. The domains were aligned up or down by applying +8, −8, +8, and −8 V after the 7 × 7 μm^2^ region was separated into 4 areas in the transverse direction. Then, a lock-in amplifier was used to apply an AC signal to a region 10 × 10 μm^2^ larger than the region where the domain was initially aligned. Figure 1c shows the amplitude (left, upper panel) and phase (left, lower panel), in accordance with the differently aligned domain directions. This enabled the confirmation of the switchable polarization domain. Next, PFM spectroscopy measurements were performed to verify the hysteresis switching. The voltage was swept between −7 and +7 V, while an AC signal was applied with the lock-in amplifier at a frequency of 13 kHz, phase of 40°, and amplitude of 2 V. In Figure 1c, the hysteresis loop caused by the piezoelectrics and/or polarization can be observed in the amplitude data (right, upper panel). The polarization is aligned upward or downward in the phase data (right, lower panel), resulting in a phase difference of 180°. This indicates that the HZO thin film has ferroelectric properties that maintain polarization

In general, Hafnia-based thin films have a multidomain structure with a polycrystalline structure. It is well known that the domains are switched over a specific range of electric fields during a certain time according to numerous mechanisms, and previous models have examined these domain transitions [18,19]. Owing to these properties, the polarization reverse rate has a particular distribution, as can be explained by the parallel resistance model [13].

(1) $$\frac{1}{R}=\frac{\left(1-S\right)}{R_{\mathrm{LRS}}}+\frac{S}{R_{\mathrm{HRS}}}$$

Here, R is the current state of resistance, R_LRS_ is the lowest resistance state (ON state) due to the upwardly aligned polarization, and R_HRS_ is the highest resistance state (OFF state) due to the downwardly aligned polarization. S is the polarization state (fully upward, S = 0; fully downward, S = 1). A nucleation-limited switching model based on the domain nucleation process is effective for describing polarization reversal in polycrystalline hafnia-based thin films [20].

(2) $$S=\int_{-\infty }^{+\infty }\left\{1-\exp\left[-{\left(\frac{t}{t_{0}}\right)}^{n}\right]\right\}\times F\left(\log t_{0}\right)d\left(\log t_{0}\right)\;$$

Here, n is the effective dimension, *t_0_* is the mean switching time, and F(log t_0_) is the Lorentz distribution.

(3) $$F\left(\log t_{0}\right)=\frac{A}{\pi }\times \left[\frac{w}{{\left(\log t-\log t_{0}\right)}^{2}+w^{2}}\right]\;$$

In the above equation, A is a normalization constant, and w is the half-width at half-maximum. The polarization reverse rate can be accurately modified in accordance with this Lorentz distribution, depending on the amplitude and width of the applied pulse.

The switching characteristics of the FTJ originate from the tunneling mechanism [21]. There are three current transport mechanisms in the FTJ, and other transport mechanisms can play major roles, depending on the voltage range and thickness of the ferroelectric layer. The three mechanisms of electron transport in the FTJ are thermionic injection, direct tunneling, and Fowler–Nordheim tunneling (FNT). These have been analyzed based on experimental data. The primary mechanism is FNT, because the FTJ is driven by the difference in the tunneling current resulting from the asymmetric potential barrier along the polarization direction.

**Figure 2:**
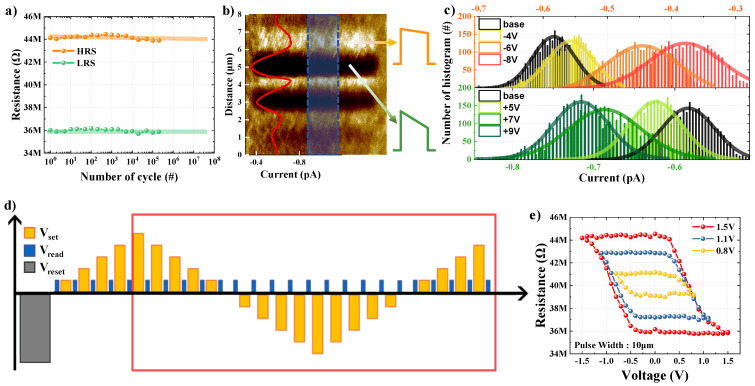
Verification of reproduced tunnelling electro-resistance (TER) effects and analog operating characteristics of HZO FTJ devices. (**a**) TER effects verification and reproducibility confirmation. (**b**) C-AFM image of the HZO/TiN structure. The inset data (red line) is the current mapping characteristics. The orange energy band diagram (EBD) corresponds to the high-resistance state (HRS), and the green EBD corresponds to the low-resistance state (LRS). (**c**) Current distributions of regions with different polarities and amplitudes of applied voltages: The HRS (right and upper panels) and LRS (right and lower panels) are the results from negative and positive voltages, respectively. (**d**) The sequences for the measurements of the R-V hysteresis loops with amplitude. (**e**) R–V hysteresis loops as function of pulse amplitude.

Asymmetric potential barriers must exist at the interfaces of the top and bottom electrodes for them to operate as FNT mechanisms. Ideally, the potential barrier should be symmetrical, because the FTJ produced in this study uses TiN for both the top and bottom electrodes. However, TiN may be partially oxidized by the reactant gas O_2_ when HZO is deposited on the bottom electrode using ALD, and a TiO_x_N_y_ layer may form at the interface [22,23]. As a result, an asymmetric potential barrier is created by the differing screen lengths at the top and bottom electrode interfaces. Owing to the asymmetric potential barrier, a state in which a significant amount of current flows due to low energy barriers to tunneling is called a low-resistance state (LRS); in contrast, a state in which less current flows due to high energy barriers to tunneling is called a high-resistance state (HRS). The tunnelling electro-resistance effect is a phrase for describing these two electrical resistance states [24]. In this study, the HRS and LRS of the manufactured FTJ device were verified using a semiconductor parameter analyzer (Figure 2a). A reading voltage of −0.2 V was used to extract the LRS and HRS currents after applying pulses with an amplitude of +1.5 V and a width of 100 μs and an amplitude of −1.5 V and a width of 100 μs, respectively. When the + voltage was applied, the LRS was confirmed, and when the – voltage was applied, the HRS was confirmed. These states were maintained well, even for repeated measurements at iterations of 10^5^ or more.

Current mapping was performed in the C-AFM mode to further verify the FTJ characteristics. C-AFM can extract the current distribution by scanning with a conductive cantilever contacting the sample to obtain information on the topography and to measure the current between the cantilever and sample. A high-performance ultra-low current amplifier (ULCA, Park Systems) with noise in units of fA was utilized, because the current level measured in this experiment was very low, on the order of a few pA. Notably, the C-AFM mode applies bias to the bottom electrode, in contrast to the PFM mode, which applies a tip bias corresponding to the top electrode. First, the domain was aligned by dividing the 6 μm × 6 μm area into 6 zones in the transverse direction and applying −4, +5, −6, +7, −8, and +9 V from the bottom based on the tip. Subsequently, the 8 μm × 8 μm area, that is, larger than the area where the domain was first aligned, was scanned to compare the currents in various states of regions and in the pristine state. Figure 2b indicates the mapped currents in color, and the current levels in the areas where negative and positive voltages are applied differ from those in the pristine state. As shown in the red line indicating the current value in Figure 2b, it can be seen that less current flows in the area where a negative voltage is applied, and more current flows in the area where the positive voltage is applied; this tendency is the same as that shown in Figure 2a. Figure 2c indicates the current distribution as mapped to various voltages extracted from the red line in Figure 2b. The upper panel indicates the result from applying the negative voltage corresponding to the HRS, and the lower panel indicates the result from applying the positive voltage corresponding to the LRS. The current distribution is biased toward the lower current in the area where the negative voltage is applied, and a larger current is applied in the area where the positive voltage is applied relative to that in the pristine area. All results have a specific distribution rather than a single value. This is expected to result in a specific coercive field, because the switching voltage is different for each domain. A normal distribution was obtained by applying the measured current distribution to a Gaussian function. The average current values in the region where −4, −6, and −8 V are applied are −0.57, −0.46, and −0.42 pA, respectively, and those where +5, +7, and +9 V are applied are −0.64, −0.69, and −0.73 pA, respectively. This indicates that the current deviates more from −0.61 pA (the pristine state) when a large voltage is applied. Furthermore, the peak current values in the region where −4, −6, and −8 V are applied are −0.55, −0.45, and −0.38 pA, respectively, and the peak current values in the region where +5, +7, and +9 V are applied are −0.63, −0.69, and −0.72 pA, respectively. It is clear that the current is distributed based on the dominant current according to the applied voltage, as the peak current is approximately the same as the average current. In other words, the various states between the HRS and LRS can be implemented by modifying the applied voltage.

The multiple resistance states between the HRS and LRS were examined considering the characteristics of the domain with a particular switching distribution. To induce polarization switching, a simple rectangular pulse with a width of 10 μs was utilized. In addition, the maximum voltage (V_max_) and minimum voltage (V_min_) of the programming pulse amplitude range were set to +0.8, +1.1, and +1.5 V and −0.8, −1.1, and −1.5 V, respectively. The measuring sequence is depicted in Figure 2d. Before the measurement, a pulse with an amplitude of −1.5 V and a width of 100 μs was applied in advance to create a HRS. Thereafter, the programming pulse was applied to increase by 0.1 V from 0 V to V_max_, and then to decrease by 0.1 V from V_max_ to V_min_, and, finally, to increase by 0.1 V from V_min_ to V_max_. The current was extracted using a reading voltage of −0.2 V in between programming pulses. The resistance was obtained by dividing the reading voltage by the extracted current. As shown by the red line in Figure 2d, only the resistance values from V_max_ to V_min_ and from V_min_ to V_max_ were plotted to assess only the dynamic range according to the amplitude range. Figure 2e indicates that the resistance state can be accurately adjusted by partially switching the polarizations of the HRS and LRS. Based on this analog operation, an experiment was conducted to imitate and control the biological synaptic plasticity, based on memristor characteristics [13,25] through a multi-resistance state.

**Figure 3:**
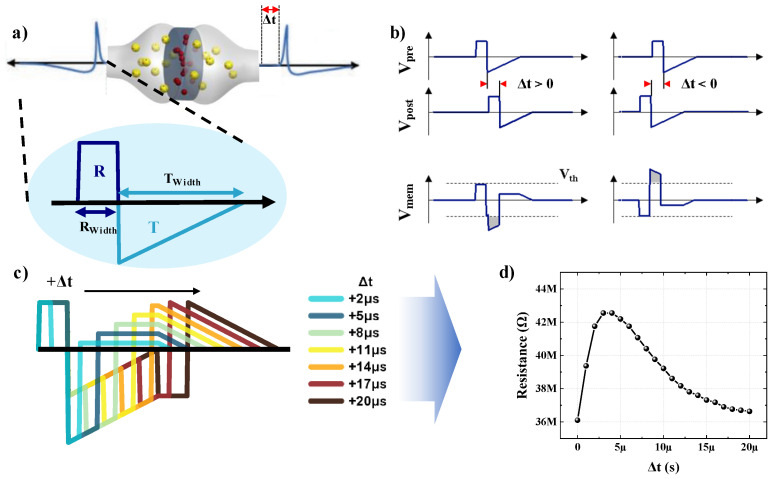
Schematic representation of the imitation of the biological synapse of a memristor device: STDP. (**a**) Concept of STDP measurement for the neuron–synapse–neuron in a memristor device (upper), and the measurement-related spike shape (lower). (**b**) Ideal model of STDP measurement for memristor device. (**c**) Time-dependent properties of programming spikes. (**d**) Resistance state modulation using the STDP learning method.

Generally, the process of signal transmission between neurons in the human brain reflects transient differences or causal relationships, and the intensity of this signal depends heavily on the synapse activity. The STDP learning method is considered similar to the operation method of the human brain, because it adjusts its strength according to the close temporal correlations between the spikes of pre- and post-synaptic neurons. This enables the implementation of long-term potentiation (LTP) and long-term depression (LTD); these are key design elements of neuromorphic computing for performing functions, such as learning, memory, and computing [8]. In an artificial synapse device, the synapse weight can be adjusted by the superposition of the voltage signals applied to both ends of the synapse (Figure 3a, upper panel). It is very important to ensure the diversity of the overlapping signals, owing to the time differences of the signals applied to the synapse. This indicates that the outcome of the STDP learning is significantly influenced by the setting of the spike applied to the synapse device. The spike-setting conditions for the STDP characteristic extraction in the HZO-based FTJ device were as follows. First, the spike corresponding to the pre- and post-neuron waveforms can be described as a concatenation of signals with different polarities, generally in the form of two continued pulses. The STDP was studied based on virtual memristor with a clear threshold voltage (±V_th_) [26]. Ideally, when there is no temporal correlation between pre- and post-spikes, the memory conductivity should not change, because a single spike cannot exceed the threshold voltage; instead, the conductivity should change when the pre- and post-spikes overlap. For this reason, the conductance change caused by a single spike should be kept to a minimum, whereas the change caused by a combination of pre- and post-spikes should be maximized beyond the threshold. Accordingly, in this study, the maximum amplitude of a single spike was reduced by using two pulses for constituting the spike with the same amplitude. Additionally, as indicated in Figure 2e, the amplitude of a spike was set to 0.75 V, because the device was in a HRS/LRS in a pulse with an amplitude of ±1.5 V.

Typically, the shape of the pulse constituting a spike has the following shapes: EE, RE, TT, RT, and RR (E: Exponential, R: Rectangular, T: Triangle) [27]. Here, a rectangular pulse (R) with a persistent amplitude was chosen as the first pulse, because sufficient time is required to change the conductivity through polarization switching. The following pulse used a triangle pulse (T), in which the voltage linearly decreased during a specific width, to realize a uniform large number of states, as this is the most important condition in the synapse device. When two pulses overlap according to timing, that is, Δt, it is anticipated that the overlapping pulses with a sufficient duration (by R) and linearly varying voltage amplitude (by T) can be obtained. That is, a RT spike was set in which R and T were continuously held at an amplitude of +0.75 and −0.75 V, respectively (Figure 3a, lower panel). Figure 3b illustrates when the RT spikes overlap. If Δt is larger than 0, the T of the pre-spike and R of the post-spike overlap exceed −V_th_ (left panel); if Δt is less than 0, the R of the pre-spike and T of the post-spike overlap to surpass +V_th_ (right panel). The overlapping spikes have different polarities and the same shapes when the absolute values of +Δt and −Δt are equal. Figure 3c depicts the overlapping signal when Δt is greater than 0 to verify the voltage signal in accordance with the change in Δt. Applying an identical spike to the top and bottom electrodes while modifying only Δt allowed us to mimic the overlaps of spikes applied to both ends of the synapse. As Δt changes, different signals occur, owing to the alterations in the amount of overlap between the T of the pre-spike and the R of the post-spike. The multiple-resistance state of the device according to the overlap of the spikes was extracted by increasing Δt by 1 μs (Figure 3d). This meant that our FTJ device could imitate and control the timing-dependent synaptic plasticity.

For our FTJ device to obtain excellent synaptic characteristics, the spike condition was suitably set by adjusting the widths of R and T. Measurements were made in the following order. When Δt was larger than 0, a pulse with an amplitude of +1.5 V and duration of 100 μs was applied in advance to ensure that the polarization was always downward (LRS) during every measurement. When Δt was less than 0, a pulse with an amplitude of −1.5 V and duration of 100 μs was applied in advance so that the polarization was always upward (HRS). Pre- and post-spikes were then applied in accordance with Δt as Δt changed from 0 to the end of the region where the pre- and post-spikes overlapped. The interval at Δt > 0 was fixed to +1 μs, and the interval at Δt < 0 was fixed to −1 μs. The conductance (G) was calculated by using the reciprocal of the resistance measured at −0.2 reading voltage, and ΔG, which was acquired based on the time Δt was 0, was plotted in accordance with Δt.

**Figure 4:**
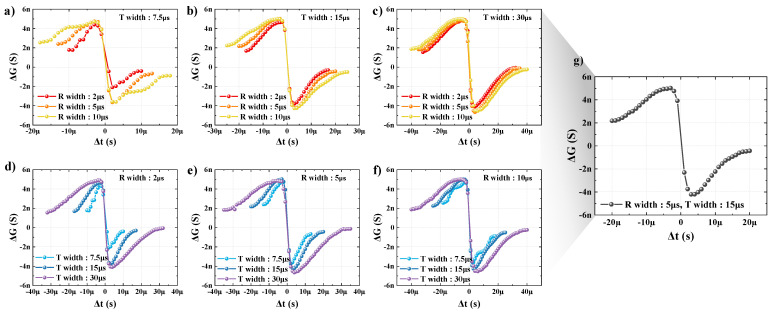
Multiple conductance characteristics with STDP Learning. (**a**–**c**) ΔG–Δt graph as function of R. (**d**–**f**) ΔG–Δt characteristic curve as function of T. (**g**) ΔG–Δt characteristic curve of optimized spike condition.

Figure 4a–c indicates the effects of R for different widths of T, that is, 7.5, 15, and 30 μs. In Figure 4a, where T is fixed to 7.5 μs, the number of controllable middle states when the width of R is 2 μs is reduced, and when the width of R is 10 μs, it can be seen that ΔG is non-linear. Additionally, it can be observed in Figure 4b,c that where the width of T is wider than it is in Figure 4a, the differences in the number of middle states and non-linearity according to the width of R are not significant. This means that it is clear that the number of middle states that can be implemented varies with the condition of R and is influenced by the condition of T. Figure 4d–f indicates the effects of T for different R widths of 2, 5, and 10 μs. In Figure 4d–f, it is clear that a short width of T is disadvantageous to the number of middle states. As a result, we determined that a spike with R = 5 μs and T = 15 μs is the most suitable by considering the entire width of the spike, as well as the multiple states from R and linearity from T (Figure 4g). It was confirmed that the synaptic characteristics (potentiation and depression) could be precisely adjusted according to the shape of the spike.

**Figure 5:**
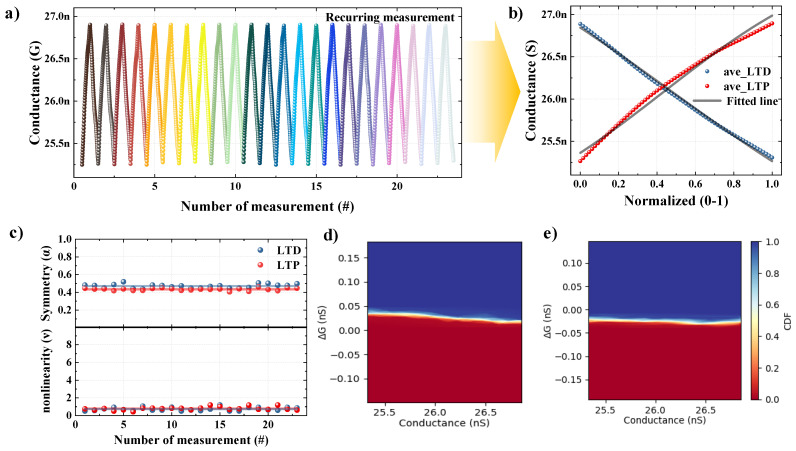
Synaptic characteristics using an optimized spike condition. (**a**) Long-term potentiation (LTP) and long-term depression (LTD) over 23 cycles with optimized spike conditions. (**b**) Averaged and normalized LTP and LTD fitted to the symmetric non-linear model. (**c**) Individual symmetric center points (upper panel) and non-linearity (lower panel) of LTP and LTD over 23 cycles. Conductivity deviation of (**d**) LTP and (**e**) LTD as statistically calculated by applying the cumulative distribution function.

We studied the synaptic characteristics of the LTP and LTD to verify the applicability of our HZO FTJ device as an artificial synapse. Figure 5a indicates the ΔG–Δt characteristic curve obtained from 23 repeated measurements using the spike in the shape optimized as indicated in Figure 4g. The LTP and LTD characteristic curves were extracted from 23 times of repeated measurements in the range of 25.3 to 26.8 nS. In an artificial neural network simulation, such parameters as linearity, symmetry, and conductance state are considered to result in high learning acuity for pattern recognition. These parameters can be characterized according to the changes in the synaptic weight as asymmetric, symmetric, linear, or non-linear. A symmetric non-linearity model [28] was used among the synapse weight change models to evaluate the linearity and symmetry of the LTP and LTD characteristic curves. The functions for p (normalized measurement number) and G (conductance) are shown in Equations (4)–(6).

(4) $$G=A\times \frac{1}{1+\exp\left[-2v\left(p-\alpha \right)\right]}+B\;$$

(5) $$A=\left(G_{\max}-G_{\min}\right)\times \frac{\exp v+1}{\exp v-1}\;$$

(6) $$B=G_{\min}-\frac{\left(G_{\max}-G_{\min}\right)}{\exp v-1}\;$$

In the above equation, G_max_ and G_min_ are the maximum and minimum conductance on the LTP and LTD characteristic curves, respectively. ν is a parameter (0 ≤ ν ≤ 10) for characterizing the non-linearity of the LTP and LTD curves, with values closer to 0 indicating ideal linear characteristics. α is the symmetric center point of the LTD and LTP characteristic curves, with values closer to 0.5 indicating ideal symmetric characteristics. Figure 5b shows the fitted line obtained by applying Equation (4) to the mean LTP and LTD curves. The LTP characteristic curves were extracted with α = 0.43 and *v* = 0.98, and the LTD characteristic curves were extracted with α = 0.47 and *v* = 0.65. The fitted line agrees with the synaptic weights of the HZO synaptic device. Figure 5c shows the characteristics of the individual LTP and LTD characteristics to confirm the reproducibility. The excellent reproducibility of the synaptic characteristics can be confirmed from the symmetric and non-linearity parameters of the 23 individual measurements, with both having values close to the average. Figure 5d,e show the conductance deviations of the probability-based, statistically calculated LTP and LTD characteristic curves, respectively. The conductance deviation was extracted by applying the amount of change in the conductance state to the probability distribution function. It was confirmed that the ΔG values of the LTP and LTD are almost comparable, and that the conduction deviation is small. Through this, it was demonstrated that excellent symmetric and linear synaptic properties can be extracted from the optimized spike.

**Figure 6:**
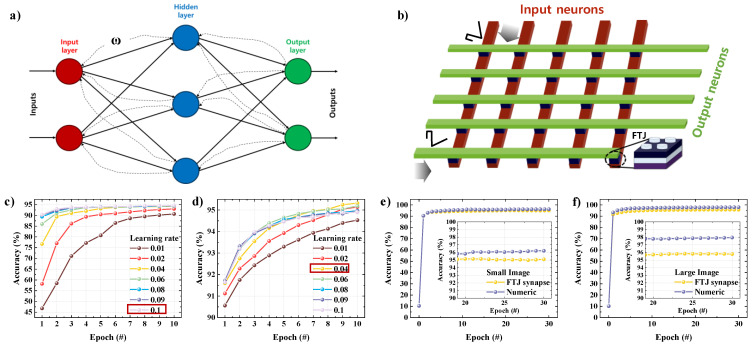
Artificial neural network simulation based on the LTP and LTD data extracted from the HZO FTJ device. (**a**) Schematic representation of artificial neural network used for the recognition of handwritten digit images used in the simulation. (**b**) Schematic representation of the neural core of the crossbar structure used in the simulation. Pattern recognition accuracy for small-image test datasets (**c**), large-image dataset (**d**) as a function of learning rate. Pattern recognition accuracy of the HZO FTJ device for the small-image test datasets (**e**) and large-image test datasets (**f**).

We performed an artificial neural network simulation using the CrossSim platform offered by Sandia National Laboratories, based on the data extracted from the spike conditions optimized for our FTJ devices. As shown in Figure 6a, the neural network architecture consisted of three layers, input/hidden/output, and the circular part of the network was called a neuron or node. In an artificial neural network, the error of the neural network is greatly influenced by the weights and is calculated based on the value input to the input layer and the expected value of the output. Learning denotes the process or algorithm for calculating errors in this neural network and correcting the weights to minimize them. CrossSim performs simulations based on backpropagation algorithms. The weight was updated to the optimal weight in proportion to the learning rate. Figure 6b is a schematic diagram of a neural core as configured in a crossbar simulator. Between the input and hidden layers, the input layer corresponds to the input neuron and the hidden layer corresponds the output neuron. Similarly, between the hidden and output layers, the hidden layer corresponds to the input neuron and the output layer corresponds to the output neuron. In the hidden layer, a weight is added to the received data, and the result is applied to the activation function again to generate the output. In this study, instead of utilizing simulation values, the weights for the simulator were determined by using values created based on experimentally extracted data. A value within a particular distribution was given a random initial weight, and simulation was performed by reflecting the random noise generated during the weight modification. To evaluate the pattern recognition accuracy, we used a small handwritten image dataset (8 × 8 pixels) provided by UCI and a large handwritten image dataset (28 × 28 pixels) provided by MNIST. The small- and large-image datasets had training datasets with 3823 and 60,000 images and test datasets with 1797 and 10,000 images, respectively.

For every 60,000 (3823) images learned, a large image (small image) was considered to have developed for one epoch, and each epoch’s accuracy was evaluated with 10,000 (1797) brand-new images that did not intervene in the learning process. The pixel brightness in the UCI and MNIST handwriting image datasets corresponds to a value between 0 and 1, and these values were input to the neuron of the input layer, respectively. The UCI and MNIST datasets contain 28 × 28 and 8 × 8 pixels, respectively, and the numbers of input layers were determined as 784 and 64, respectively, and hidden layers were determined as 36 neurons and 300 neurons, respectively. The output layer contained 10 neurons because it represented a number represented by the UCI and MNIST handwritten images. The output layer produced all results associated with the data input through the input layer.

We optimized the learning rate for an accuracy evaluation of the pattern recognition, because the weights were updated relative to the learning rate. The learning rate was optimized between 0.01 and 0.1 on the small- and large-image datasets over 10 epochs (Figure 6c). The small-image dataset is saturated at approximately 94% accuracy on roughly 6 epochs when the learning rate is 0.04 or above; however, when it is less than 0.04, it results in a lower accuracy. In addition, when the learning rate is 0.1, the accuracy increases the fastest and saturates. The large-image datasets indicate the fastest improvement in accuracy when the learning rate is 0.09, but the highest accuracy is shown at 10 epochs, when the learning rate is 0.04 (Figure 6d) Accordingly, the learning rates for the small-image and large-image datasets were set to 0.01 and 0.04, respectively. Figure 6e,f shows the accuracy of pattern recognition at up to 30 epochs when the optimized learning rates are applied to the small- and large-image datasets. The small- and large-image datasets have 95.10% and 95.79% accuracy at 30 epochs, respectively, that is, slight differences of just 1.11% and 2.16% from the ideal accuracies, respectively. The high accuracy of the pattern recognition is based on the synaptic characteristics, such as excellent symmetry, linearity, and reproducibility. Thus, our FTJ device successfully controls the synaptic characteristics through spike shape control.

## 4. Conclusions

In conclusion, we demonstrated the potential of an HZO FTJ in SNN-based synaptic devices for neuromorphic computing. The HZO ferroelectric 5 nm-thin film was deposited using PEALD to create an FTJ device. The excellent ferroelectricity of the manufactured FTJ was confirmed through the typical butterfly curve characteristics through AFM and the polarization domain of 180°. Based on its excellent ferroelectric properties, the operating characteristics of the FTJ device were investigated, and it was confirmed that multiple resistance states could be controlled through the analysis of the C-AFM current distribution and R-V hysteresis loop. The shape of the spike affecting the STDP learning was optimized, and the synaptic characteristics of the LTP and LTD were measured. Repeated measurements revealed synaptic properties with high symmetry (α = 0.43 & 0.47), linearity(ν = 0.98 & 0.65), and reproducibility. An artificial neural network simulation using a CrossSim simulator was conducted with the extracted LTP and LTD data. Pattern recognition simulated using the UCI and MNIST datasets demonstrated high levels of accuracy (95.10% and 95.79%, respectively). These results indicate that our HZO FTJ device is an excellent candidate for an efficient SNN synaptic device, and that it has strong potential for neuromorphic computing applications.

## Data Availability

The data presented in this study are contained within the article.
